# A single-dose, randomized, open-labeled, parallel-group study comparing the pharmacokinetics, pharmacodynamics and safety of leuprolide acetate microspheres 3.75 mg and Enantone^®^ 3.75 mg in healthy male subjects

**DOI:** 10.3389/fphar.2022.946505

**Published:** 2022-08-19

**Authors:** Xingjiang Hu, Qiao Zhang, Yunliang Zheng, You Zhai, Nana Xu, Qingwei Zhao, Jian Liu, Longyan Wan, Jindan Luo

**Affiliations:** ^1^ Research Center for Clinical Pharmacy, Zhejiang Provincial Key Laboratory for Drug Evaluation and Clinical Research, The First Affiliated Hospital, School of Medicine, Zhejiang University, Hangzhou, China; ^2^ Shanghai Livzon Pharmacy Co., Ltd, Shanghai, Zhejiang, China; ^3^ Urology Surgery, The First Affiliated Hospital, School of Medicine, Zhejiang University, Hangzhou, Zhejiang, China

**Keywords:** leuprolide, comparative study, pharmacokinetic, pharmacodynamic, microsphere

## Abstract

Leuprolide acetate microspheres developed by Shanghai Livzon Pharmaceutical Co., Ltd. (T) have been marketed in China for more than 10 years, benefiting a large number of patients, and will continue to play an important role in China. However, as a generic drug, it is unclear whether there is a difference in efficacy between T and the original product Enantone^®^ (R). This study compared the differences in efficacy and safety of two 1-month depot formulations in 48 healthy Chinese male subjects by comparing multiple pharmacokinetic (PK) and pharmacodynamic (PD) parameters. The main research indicators were the PK parameters of leuprolide (C_max_, AUC_0-t_, AUC_0-D7_, and AUC_D7-t_) and the PD parameters of testosterone (E_max_, AUEC_0-t_, AUEC_0-D7_, and AUEC_D7-t_) after 42 days of administration. The C_max_, AUC_0-t_, AUC_0-D7_ and AUC_D7-t_ of leuprolide were slightly higher in the T group than in the R group with 90% confidence intervals (CIs) of 94.43–118.53%, 109.13–141.88%, 109.53–139.54%, and 105.17–145.74%, respectively. No significant differences in the PD parameters (E_max_, AUEC_0-t_, AUEC_0-D7_, and AUEC_D7-t_) existed between the T and R groups, and 90% CIs were 62.80–93.57%, 88.17–110.55, 95.72%–118.50%, and 79.77–105.63, respectively. At 672 h (D28), the castration rate of T was 91.30% (21/23) and that of R was 60.87% (14/23). The PK characteristics were consistent and the inhibitory effects on testosterone levels were similar in both T and R groups; further, clinical safety was observed for both T and R formulations, suggesting that these two products can replace each other in clinical practice.

**Clinical Trial Registration:** http://www.chinadrugtrials.org.cn/clinicaltrials.searchlistdetail.dhtml, identifier CTR20200641.

## Introduction

Gonadotropin-releasing hormone (GnRH) agonists (e.g., leuprolide acetate, goserelin acetate, and triptorelin) or antagonists can suppress the release of gonadotropins, such as luteinizing hormone (LH) and follicle-stimulating hormone (FSH), and subsequently inhibit the production of estradiol and testosterone (Geiges et al., 2013). GnRH agonists are common preoperative therapies for a variety of diseases, including endometriosis, uterine myoma, premenopausal breast cancer, prostate cancer, and central precocity (Barbieri, 1992; Park et al., 2017). They can produce an initial transient increase in gonadotropin levels, called the flare effect. Continuous GnRH agonist therapy could induce downregulation and desensitization of GnRH receptors, producing a hypogonadotropic state and inhibiting pituitary gonadotropin production and release (Dragnic et al., 2016). It could inhibit ovarian and testicular responses to gonadotropin, thereby reducing the production of estradiol and testosterone (chronic effects).

Leuprolide acetate is a type of GnRH agonist that is commonly used for treating patients with endometriosis, uterine myoma with menstruation, abdominal pain, back pain, anemia, premenopausal breast cancer, estrogen receptor-positive prostate cancer, and central precocity (Yasukawa et al., 2005). The LH-releasing activity of leuprolide acetate is approximately 100 times that of natural GnRH, and its inhibitory effect on the pituitary-gonadal system is stronger than that of natural GnRH. Because its resistance to proteolytic enzymes and affinity to the GnRH receptor are stronger than those of endogenous GnRH, it can effectively inhibit the function of the pituitary-gonadal system. The pharmacological mechanisms of leuprolide indicate that it needs to be maintained at a relatively stable level for prolonged periods in order to be effective. Because repeated administration of leuprolide increases the burden on patients and reduces their quality of life, new preparations of 1-month, 3-month, and 6-month depot formulations, or even longer, have been successfully developed and applied in clinics (Suzuki et al., 2015).

Leuprolide microspheres are a type of special injection formulation with complex sustained-release behavior, and are prepared by embedding polylactic-co-glycolic acid (PLGA) with leuprolide. Owing to the uneven and heterogeneous degradation rate of PLGA, uneven release and fluctuation in the blood concentration of leuprolide have been observed. Leuprorelin acetate microspheres developed by Takeda Pharmaceutical Industry Co., Ltd. (Enantone ^®^) for injection were approved by the Food and Drug Administration in 1989 and the China Food and Drug Administration (CFDA) in 2003 (Sartor, 2003). Since then, thousands of patients with endometriosis, central precocious puberty, breast cancer, and prostate cancer have benefited from it, and good clinical efficacy has been achieved. As a generic product, leuprorelin acetate microspheres, developed by Shanghai Livzon for injection, were approved by the CFDA in 2009; their market share has been increasing, benefiting large groups of patients in China. However, it is not clear whether there are differences in the pharmacokinetic (PK) and pharmacodynamic (PD) parameters between this product and Enantone^®^. A similar study of leuprorelin acetate microspheres for injection produced by Zhaoke in China found that the PK parameters of their products were consistent with those of Enantone^®^. Similar inhibitory effects on testosterone and a longer castration time were maintained for their products than for Enantone^®^ (Zhou et al., 2021). Our research provides richer and more complete data to compare the PK and PD parameters of leuprolide acetate microspheres (3.75 mg, 1-month) produced by Shanghai Livzon with those of Enantone^®^ (3.75 mg, 1-month).

## Materials and methods

### Inclusion and exclusion criteria

According to our clinical trial protocol (No: YYL-LB-007, V2.0), healthy male subjects aged 18–40 years, with body weights of no less than 50.0 kg and body mass index (BMI) between 19.0 and 26.0 kg m^−2^, were considered eligible to participate in this study. Before the initiation of screening, written informed consent was obtained from every subject after informing them about the study objectives, procedures, and possible risks. The health status of the subjects was determined by recording their medical history, demographic data, and data acquired from physical examination, 12-lead electrocardiogram (ECG) tests, abdominal ultrasound scan, chest X-ray, and hematology laboratory tests during the screening period (2 weeks before the day of leuprolide acetate microspheres drug administration). All subjects were required to take effective contraceptive measures in the following 6 months.

The exclusion criteria were: allergy to any component of leuprolide acetate microspheres for injection, GnRH analogs, or allergic physique (allergy to two or more drugs, food, and pollen); medical history of any cardiovascular disease, pulmonary disease, kidney disease, diabetes, anemia, dysphagia; any history of gastrointestinal disease that could affect drug absorption; or history of uncontrolled peptic ulcer, colitis, or pancreatitis. Subjects with a history of alcohol, nicotine, or drug abuse and positive reactions for human immunodeficiency virus, hepatitis B virus surface antigen, anti-hepatitis C virus antibody, or syphilis antibody were also excluded. Other exclusion criteria included abnormal sex hormone levels, blood donation within the previous 3 months, participation in other drug trials within the previous 3 months, and drug treatment in the previous 2 weeks.

### Study design and treatment

**FIGURE 1:**
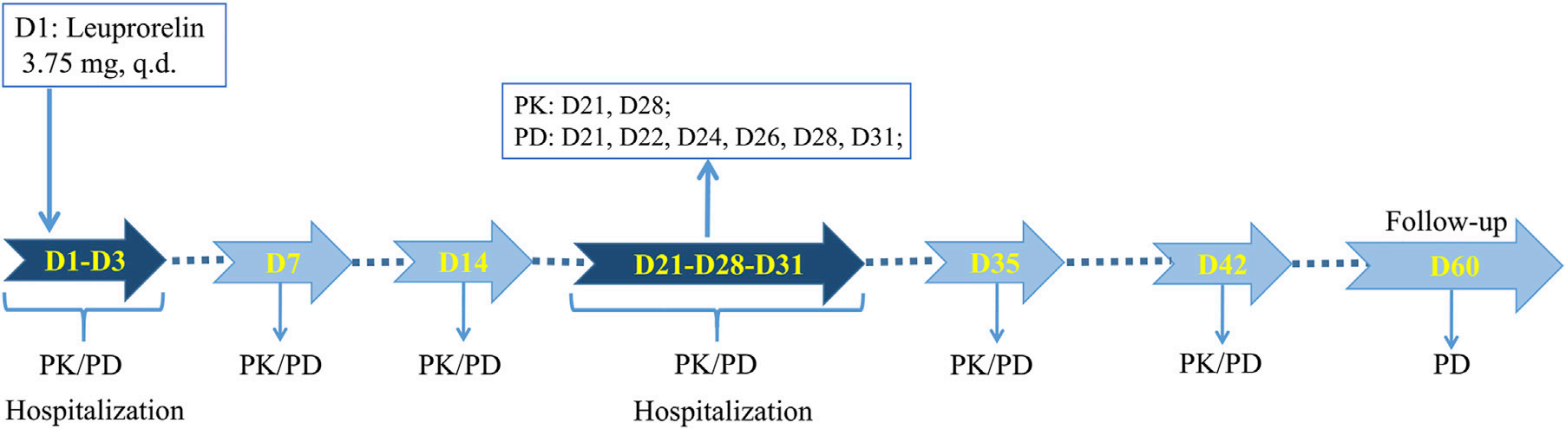
Flow chart of clinical trial design.

This study was approved by the Ethics Committee of the First Affiliated Hospital, College of Medicine, Zhejiang University (2019-162). As a single-dose, randomized, open-ended, parallel-group comparative investigation, this study was registered on chinadrugtrials. org.cn (CTR20200641). Eligible subjects were randomly assigned to two groups of leuprolide acetate microspheres developed by Shanghai Livzon Pharmaceutical Co., Ltd. (T) and Takeda Pharmaceutical Industry Co., Ltd. (R) in a 1:1 ratio and were subcutaneously injected with corresponding microspheres on an empty stomach. The trial process is shown in Figure 1, where the subjects were required to cooperate with the researchers for PK and PD blood collection, or safety inspection at the following time points: 1) One day before administration (D1), the subjects were admitted to the phase I ward of the First Affiliated Hospital, School of Medicine, Zhejiang University, for unified management. They were then allowed to leave the phase I ward after the completion of blood collection and safety inspection 72 h after administration (D3). 2) At 168 h (D7) and 336 h (D14) after administration, they returned to the phase I ward for PK and PD blood sample collection, and vital sign examination. 3) After administration, at 504 h (D21), they returned to the phase I ward for PK and PD blood sample collection and safety inspection and were admitted to the ward for unified management. At 744 h (D31), they were allowed to leave the phase I ward after unified management, blood collection, and vital sign examination. 4) At 840 h (D35) and 1008 h (D42) after administration, they returned to the phase I ward for PK and PD blood sample collection, and vital sign examination. 5) At last, PD blood samples and vital signs were collected at 1440 h (D60) after administration due to safety concerns, and all subjects were discharged after a safety inspection.

### Blood sampling and analysis

Blood samples were collected at 0 h (within 1 h before administration), and 0.5, 1.0, 1.5, 2.0, 2.5, 3.0, 6.0, 12.0, 24.0 (D1), 48.0 (D2), 72.0 (D3), 168.0 (D7), 336.0 (D14), 504.0 (D21), 672.0 (D28), 840.0 (D35), and 1008.0 (D42) h after administration, for PK analysis. Blood samples were collected at 0 h (within 1 h before administration), and 3.0, 6.0, 24.0 (D1), 48.0 (D2), 72.0 (D3), 168.0 (D7), 336.0 (D14), 504.0 (D21), 528 (D22), 576 (D24), 624 (D26), 672.0 (D28), 744.0 (D31), 840.0 (D35), and 1008.0 (D42) h after administration, for PD analysis. Liquid Chromatography with tandem mass spectrometry (LC-MS/MS) was used to determine the level of leuprolide in plasma and testosterone level in serum. An electrochemiluminescence immunoassay was used to determine the concentrations of FSH and LH in serum. More detailed descriptions of the quantitative analytical assays (such as linear range and lower limit of quantification) for leuprorelin, testosterone, FSH, and LH are shown in Supplementary Table S1.

### Safety assessment

All adverse events (AEs) were classified according to the organ system and preferred term used in the ICH International Dictionary of Medical Terms. The number and incidence of AEs and serious AEs (SAEs), leading to shedding in each system, were described. The number and incidence of AEs in each system were described according to severity. The results of laboratory tests, including those of blood routine, urine routine, blood biochemistry, fasting blood glucose, coagulation function, thyroid function, urine alcohol screening, drug abuse screening, and 12 lead ECG, are summarized.

### Statistical analysis

PK analysis was performed using WinNonlin 8.2 and SAS 9.4 software. The significance level of the other statistical tests was set at 0.05. For quantitative indicators, descriptive statistics included the case number, mean, standard deviation, median, minimum, maximum, and quartile. For the counting indicators, descriptive statistics included cases and percentages. The evaluation indexes were PK parameters (C_max_, AUC_0-t_, AUC_0-D7_ and AUC_D7-t_) and PD parameters (E_max_, AUEC_0-t_, AUEC_0-D7_, and AUEC_D7-t_). Tests were used to compare the differences between T and R. After taking the natural logarithm, a *t*-test was used to analyze and compare the differences between these two groups. The mean difference between T and R was calculated, and a negative value was used to calculate the geometric mean ratio and 90% confidence interval (CI) of T and R.

## Results

### Participants

As shown in Table 1, 48 male Chinese participants were enrolled. Participants had a mean age of 28.96 ± 5.86 years (range 19–40 years) and a mean BMI of 21.69 ± 1.43 kg/m^2^ (range 19.2–24.4 kg/m^2^). The mean height was 1.68 ± 0.05 m (range 1.56–1.77 cm), and the mean weight was 60.93 ± 4.04 kg (range 51.6–69.1 kg). Forty-six participants completed the study. Two subjects withdrew voluntarily after the blood samples were collected for PK analysis at 504.0 h (D21) and for PD analysis at 528.0 h (D22), respectively.

**TABLE 1:** Basic characteristics of demography.

	**T (N = 24)**	**R (N = 24)**	**In total (N = 48)**
Age			
N (Missing)	24 (0)	24 (0)	48 (0)
Mean ± SD	29.71 ± 5.33	28.21 ± 6.37	28.96 ± 5.86
Min∼Max	20∼40	19∼39	19∼40
Median	28.50	28.00	28.00
Gender			
N (Missing)	24 (0)	24 (0)	48 (0)
Female	0 (0%)	0 (0%)	0 (0%)
Male	24 (100%)	24 (100%)	48 (100%)
Nation			
N (Missing)	24 (0)	24 (0)	48 (0)
Han nationality	21 (87.50%)	24 (100%)	45 (93.75%)
Other nationalities	3 (12.50%)	0 (0%)	3 (6.25%)
Height(m)			
N (Missing)	24 (0)	24 (0)	48 (0)
Mean ± SD	1.68 ± 0.05	1.67 ± 0.05	1.68 ± 0.05
Min∼Max	1.58∼1.75	1.56∼1.77	1.56∼1.77
Median	1.69	1.66	1.68
Weight (kg)			
N (Missing)	24 (0)	24 (0)	48 (0)
Mean ± SD	61.30 ± 3.28	60.55 ± 4.72	60.93 ± 4.04
Min∼Max	55.6∼69.0	51.6∼69.1	51.6∼69.1
Median	61.25	60.50	60.95
Body mass index (kg/m^2^)			
N (Missing)	24 (0)	24 (0)	48 (0)
Mean ± SD	21.70 ± 1.21	21.69 ± 1.64	21.69 ± 1.43
Min∼Max	19.8∼24.4	19.2∼24.3	19.2∼24.4
Median	21.45	22.15	21.75

### Pharmacokinetic properties of leuprolide

**FIGURE 2:**
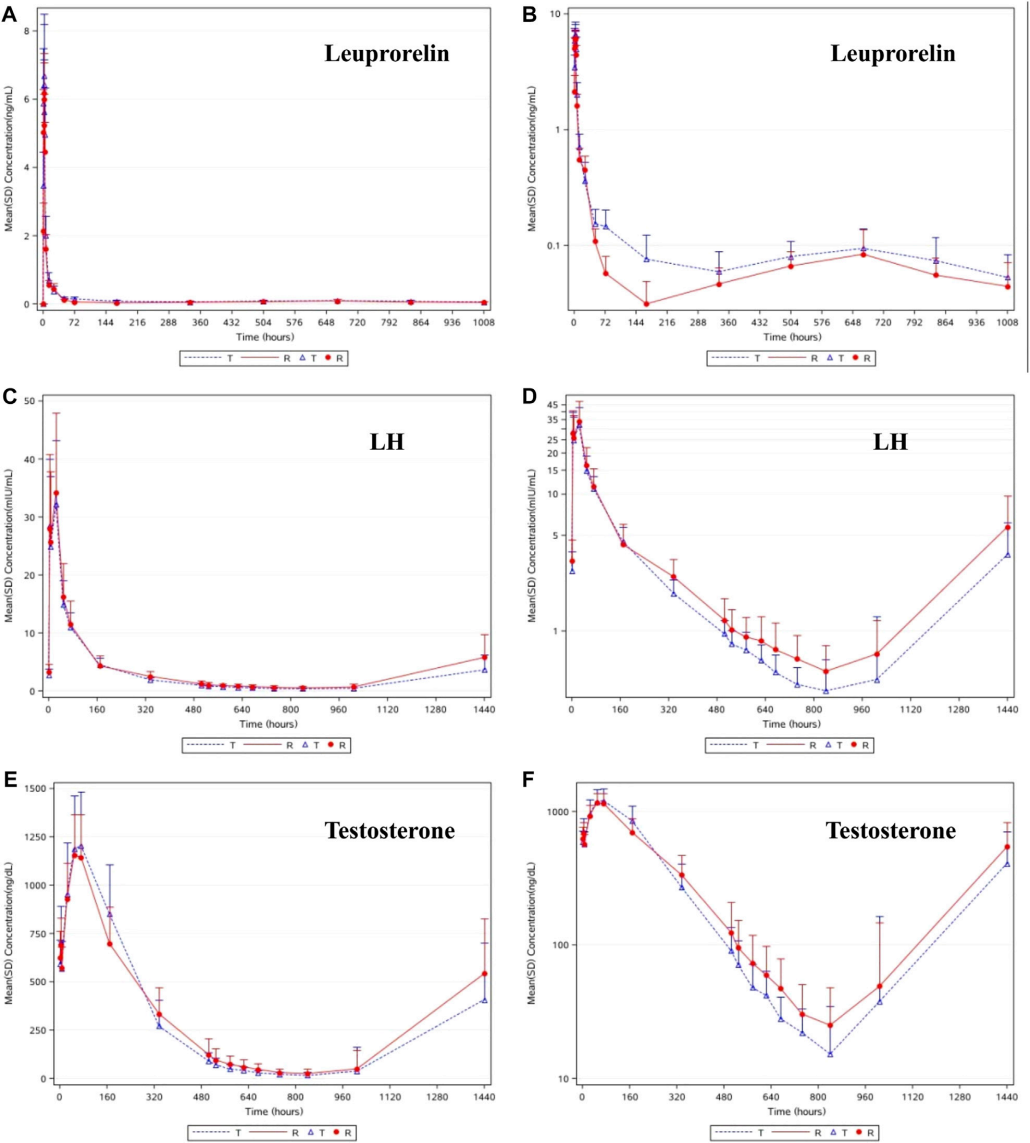
The linear **(A)** and semilog **(B)** of mean concentrations of leuprolide-time curve in serum. The linear **(C)** and semilog **(D)** of mean concentrations of LH-time curve in serum. The linear **(E)** and semilog **(F)** of median concentrations of testosterone-time curve in plasma.

As shown in Figures 2A,B, the trends in the plasma leuprolide concentrations of T and R were remarkably similar. After calculating the natural logarithm of the PK parameters, a *t*-test was used to compare the differences between the two groups. The geometric mean ratios and 90% CI of T and R are listed in Table 2. No significant difference was observed in C_max_ between T and R with a 90% CI of 94.43–118.53%. However, there were significant differences in AUC_0-t_, AUC_0-D7_ and AUC_D7-t_ where 90% CIs of the two groups were 109.13–141.88%, 109.53–139.54%, and 105.17–145.74%, respectively.

**TABLE 2:** Pharmacokinetic parameters of leuprolide: Geometric mean ratio and 90% confidence interval.

PK parameters	Geometric mean and ratio					90%CI (%)	Intra-individual variation (%)
	T		R		T/R (%)		
C_max_ (ng/ml)	*n* = 24	6.60	*n* = 24	6.23	105.79	94.43-118.53	23.79
AUC_0-t_ (h*ng/mL)	*n* = 23	116.97	*n* = 23	94.00	124.43	109.13-141.88	26.96
AUC_0-D7_ (h*ng/mL)	*n* = 24	58.24	*n* = 24	47.11	123.63	109.53-139.54	25.38
AUC_D7-t_ (h*ng/mL)	*n* = 23	58.33	*n* = 23	47.11	123.81	105.17-145.74	33.84

### Pharmacodynamic properties of LH

As shown in Figures 2C,D, after a single subcutaneous injection of leuprolide acetate microspheres in the 48 subjects, the average LH concentration increased gradually, reaching the highest level at 24.0 h (D2), then gradually decreased, reaching the lowest at 840 h (D35), and then gradually increased until 1440 h (D60), where it returned to the baseline level. Similar pharmacodynamic characteristics of LH were observed in the subjects of T and R. After the natural logarithm of the PD parameters was taken, the geometric mean ratio and 90% CI of AUEC_0-D28_ (h*mIU/mL) between T and R was 83.37% (71.52%–97.18%). No statistically significant difference in AUEC_0-D28_ of LH was observed between the two groups (*p* = 0.0525). Higher leuprolide exposure resulted in lower LH levels, indicating a good correlation between the change in LH and leuprolide levels. These results provide a reasonable explanation for the fact that the castration rate and duration were higher in the subjects of T than those of R.

### Pharmacodynamic properties of testosterone

As shown in Figures 2E,F, the change in the trend of testosterone was consistent with that of LH, and the pharmacodynamic characteristics between T and R were similar. The average testosterone concentration in the subjects gradually increased after administration of T and R preparations. The testosterone concentrations reached their highest levels at 72 h (D3) and 48 h (D2), respectively, then gradually decreased, reaching the lowest at 840 h (D35), and then gradually increased. The average testosterone concentration in the subjects of T was maintained below 50 ng/dl from 576 h (D24) to 1008 h (D42) after administration, and that of R was maintained below 50 ng/dl from 672 h (D28) to 1008 h (D42) after administration.

The main pharmacodynamic parameters of testosterone in the subjects of T and R, after a single subcutaneous injection of leuprolide acetate microspheres in 48 subjects, are summarized in Table 3. After taking the natural logarithm of the PD parameters, the geometric mean ratio (and 90% CI) of T/R were 76.66% (62.80–93.57%) for E_max_, 98.73% (88.17–110.55) for AUEC_0-t_, 106.50% (95.72%–118.50%) for AUEC_0-D7_, and 91.79% (79.77–105.63) for AUEC_D7-t_. After taking the natural logarithm, no significant difference was observed in the PD parameters of testosterone (*p* > 0.05). Considering the fact that lower testosterone levels mean longer castration time, it suggests that the efficacy of T is better than that of R.

**TABLE 3:** Pharmacodynamic parameters of testosterone: Geometric mean ratio and 90% confidence interval.

PD parameters (unit)	Geometric mean and ratio					90%CI (%)	Intra individual variation (%)
	T		R		T/R (%)		
E_max_ (ng/dl)	*n* = 23	14.21	*n* = 23	18.54	76.66	62.80-93.57	41.91
AUEC_0-t_ (h*ng/dL)	*n* = 23	305109.40	*n* = 23	309038.40	98.73	88.17-110.55	23.13
AUEC_0-D7_ (h*ng/dL)	*n* = 24	165730.16	*n* = 24	155611.83	106.50	95.72-118.50	22.29
AUEC_D7-t_ (h*ng/dL)	*n* = 23	139954.37	*n* = 23	152470.27	91.79	79.77-105.63	28.91

### Pharmacodynamic properties of FSH

**FIGURE 3:**
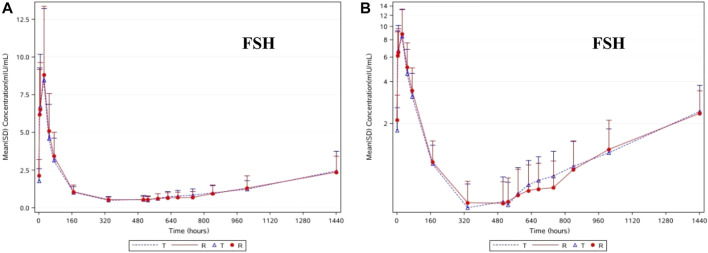
The linear **(A)** and semilog **(B)** of median concentrations of FSH-time curve in serum.

The change trends in the pharmacodynamic characteristics of FSH were similar to those of LH in the subjects of T and R. As shown in Figures 3A,B, after the administration of T and R, the average FSH concentration gradually increased, reaching its highest at 24.0 h (D2), then gradually decreased, reaching its lowest at 336 h (D14) and 504 h (D21), and then gradually increased until it returned to the baseline level at 1440 h (D60). After taking the natural logarithm of the PD parameters, the geometric mean ratio of AUEC_0-D28_ (h*mIU/mL) and its 90% CI were 98.10% and 80.89%–118.99%, respectively. No statistically significant difference in the AUEC_0-D28_ of FSH was found between these two groups (*p* = 0.8684). Unlike the consistency in leuprolide, LH, and testosterone levels, the difference in FSH levels in the subjects of T and R was relatively small.

### Safety assessment

Overall, the safety and tolerability of a single fasting injection of both kinds of leuprolide acetate microspheres were good, and there was no significant difference between T and R. Forty-eight subjects were enrolled in this study and 81 AEs occurred in 40 subjects, with an incidence of 83.33% (40/48). No SAEs or withdrawal AEs occurred. As shown in Table 4, 40 AEs occurred in 19 subjects in the T group, with an incidence of 79.17% (19/24). In the R group, 41 AEs occurred in 21 subjects, with an incidence of 87.50% (21/24). The main types of AEs in T were abnormal test parameters, gastrointestinal diseases, and hepatobiliary diseases, such as elevated triglyceride and alanine aminotransferase levels, and decreased fibrinogen levels. The AEs in R were mainly classified as abnormal test parameters, gastrointestinal diseases, and skin and subcutaneous diseases, such as neutropenia, calluses, and oral mucositis. In the T group, there was 1 case of grade 3 AEs, 3 cases of grade 2 AEs, and the other AEs were grade 1. In R, there were 3 cases of grade 2 AEs, and the other AEs were grade 1.

**TABLE 4:** Summary of all AEs after the treatment of leuprolide acetate microspheres in the subjects of T and R.

System organ class (SOC)	Disorders	T group (N = 24)		R group (N = 24)	
		Number of AEs	Subjects (%)	Number of AEs	Subjects (%)
Laboratory tests	Abnormal values	25	15 (62.50%)	22	15 (62.50%)
Gastrointestinal system diseases	Oral mucositis	2	2 (8.33%)	4	4 (16.67%)
	Diarrhea	1	1 (4.17%)	0	0 (0%)
	Abdominal pain	1	1 (4.17%)	0	0 (0%)
Skin and subcutaneous tissue diseases	Calluses	2	2 (8.33%)	3	3 (12.50%)
	Erythra	0	0 (0%)	1	1 (4.17%)
Heart organ diseases	Infranodal extrasystole	1	1 (4.17%)	1	1 (4.17%)
	Sinus bradycardia	0	0 (0%)	2	2 (8.33%)
	Sychnosphygmia	0	0 (0%)	1	1 (4.17%)
Infections	Infection of penis	0	0 (0%)	1	1 (4.17%)
	Upper respiratory tract infection	0	0 (0%)	1	1 (4.17%)
	Epididymitis	0	0 (0%)	1	1 (4.17%)
	Amygdalitis	1	1 (4.17%)	0	0 (0%)
Hepatobiliary system diseases	Abnormal liver functions	3	3 (12.50%)	1	1 (4.17%)
Various injuries, poisoning, and surgical complications	Skin abrasion	0	0 (0%)	2	2 (8.33%)
	Thermal burn	1	1 (4.17%)	0	0 (0%)
Blood and lymphoid system disorders	Lymph node pain	1	1 (4.17%)	0	0 (0%)
Kidney and urological diseases	Dysuria	1	1 (4.17%)	0	0 (0%)
Psychosis	Affective disorder	0	0 (0%)	1	1 (4.17%)
Neurological diseases	Headache	1	1 (4.17%)	0	0 (0%)
Total		40	19 (79.17%)	41	21 (87.50%)

## Discussion

Leuprolide acetate microspheres are important GnRH agonists that are commonly used worldwide, and with the progress of new formulation technology, 1-month (United States: 7.5 mg; EU and Japan: 3.75 mg) formulations have been developed, easing the burden on patients and physicians by reducing the number of injections, thereby improving the quality of life of patients (Suzuki et al., 2015). In China, there is great demand and market for these products; however, it remains unclear whether there is a difference in efficacy between the Chinese imitation product and the original product Enantone^®^. Several similar studies have been conducted in China; for example, a similar product produced by Zhaoke found that the PK parameters of their products were consistent with those of Enantone^®^. Similar inhibitory rates of testosterone and a longer castration time were maintained for their products than for Enantone^®^ (Zhou et al., 2021). The present study reported the consistency in the PK and PD parameters of the two kinds of leuprolide acetate microspheres produced by Shanghai Livzon Pharmaceutical Co., Ltd. and Enantone^®^ through reliable clinical trial data.

Compared with their research, we provided more detailed results to compare the differences between the two preparations, although our final conclusions were similar. First, we provided PK and PD data of 168 h (D7) to further compare the consistency of these two preparations. AUC_0-D7_ is associated with the fast release period (burst phase) of leuprolide; therefore, it is relevant to “flare-up.” Hence, if there is a similarity in AUC_0-D7_ in both T and R, it means a similar and sufficient binding between leuprolide and GnRH receptors. Meanwhile, the difference between the two preparations at D7 can directly reflect the possible safety difference between the two preparations. After D7, the concertation of leuprorelin in blood enters the steady-state period, and low concentrations of leuprorelin can facilitate pituitary receptor desensitization (Sartor, 2003; Eckstein and Haas, 2014; Park et al., 2017). In addition, we found that the castration rate did not reach 100%. In particular, the castration rate in the subjects of R was only 60.87% at 672 h (D28). Even at 744 h (D31), the time point of the highest castration rate, the castration rate in the R group was only 86.96%. The reason may be the differences between the preparation batches and the small sample size (number of subjects).

In our results, the resemblance of C_max_ and the curve of the steady period of leuprolide concentration in both T and R reflects the similarity of the drug release behaviors of the two formulations. The curve of T seemed smoother because it entered the plateau phase earlier and did not demonstrate a sharp valley between 144 h (D6) and 216 h (D9), similar to the reference group. The slightly higher plateau phase of leuprolide treatment resulted in better testosterone suppression. These two formulations guaranteed a similar release of leuprolide from the depot. The PK data obtained in this study for the 1-month depot are in agreement with the previously published results (Mazzei et al., 1990; Tunn et al., 2013; Zhou et al., 2021). Our results showed that changes in plasma leuprolide concentrations in the T and R depots were remarkably similar. The C_max_ of leuprolide acetate microspheres were similar between the two preparations, and the AUC_0-t_, AUC_0-D7_, and AUC_D7-t_ of T were slightly higher than those of R. Owing to the difference in AUC, it may be that the leuprolide content in the preparation produced by Shanghai Livzon was slightly higher or was affected by lymphatic circulation due to differences in particle size. However, this slight difference does not affect the efficacy, and may even achieve a better effect.

In males, LH stimulates the development of interstitial cells in the testes, which secrete testosterone (Bordini and Rosenfield, 2011). In this study, both formulations led to initial stimulation at the pituitary level, with the subsequent release of gonadotropins. However, it appears that higher exposure to leuprolide resulted in a longer time for GnRH receptor occupation and stronger inhibition of the pituitary gland, resulting in faster decline and lower concentration of LH, which was very similar to the change in the trend of testosterone levels. Thus, the change in LH, rather than FSH, appears to be the main factor affecting testosterone levels. This is related to the fact that the main function of FSH is to promote sperm formation rather than affect the concentration of testosterone. These results further suggested that LH is crucial for testosterone synthesis. In addition, stronger castration ability always indicates greater clinical benefits during clinical therapy. Therefore, at least in our study, we found that the curative effect of T is not inferior to that of Enantone^®^ and is even stronger.

Treatment with androgen deprivation therapy cannot avoid AEs such as fatigue, diminished sexual function, hot flushes, and most importantly, cardiovascular disease (Freedland and Abrahamsson, 2021). Most adverse reactions arise from dramatic fluctuations in testosterone levels. The AEs associated with Enantone^®^ (3.75 mg) monthly depot formulations include fatigue, headaches, hot flushes, hypertension, urinary retention, and local pain at the injection site (Kienle and Lubben, 1996; Geiges et al., 2013). However, few patients withdrew from the study because of AEs or SAEs, after the administration of leuprolide acetate (3.75 mg, monthly injection) (Geiges et al., 2013; Osuga et al., 2019; Zhou et al., 2021). In clinical practice, anti-androgen therapy has been used to combat the ignition effect of testosterone and minimize side effects. However, in our research, such measures could not be taken, which means that the appearance of AEs was inevitable. The main types of AEs in the T and R groups in this study were various examinations, gastrointestinal diseases, hepatobiliary diseases, and skin and subcutaneous diseases; no SAEs or withdrawal AEs occurred. The study results show that the safety of the two types of leuprolide acetate microsphere was well-tolerated with a comparable safety profile.

We provide complete and detailed data on the PK and PD characteristics of leuprolide acetate microspheres in healthy adult males and clarified the relationship between each indicator. The microspheres produced by Shanghai Livzon are an important product with a high market share in China and are one of the only two domestic products. The results of this study provide reliable evidence of the clinical substitutability of these two products; they will enrich the standardized data on GnRHa subcutaneous administration and provide a good reference for comparing the similarity of microsphere drugs. However, this study had some limitations. A sample size of 48 subjects might not be sufficient to demonstrate the equivalence of the PK parameters. In addition, the PK and PD profiles of repeated injections were not tested, and the consistency of long-term use regarding the accumulation of leuprolide and the maintenance of castration levels of testosterone were not examined in this study. However, the present data could preliminarily evaluate the possible PK and PD characteristics after multiple administrations, consistency of PK and PD changes, and similarity between these two preparations by the combined evaluation of PK and PD parameters.

In conclusion, this study showed that leuprolide acetate microspheres for injection (3.75 mg, 1-month, Shanghai Livzon Pharmaceutical Co., Ltd.) exhibited PK and PD characteristics similar to those of Enantone^®^ (3.75 mg, 1-month, Takeda Pharmaceutical Industry Co., Ltd.). Meanwhile, higher castration rates and longer castration times were observed with this domestic product than with Enantone^®^. Both preparations showed good tolerance, and there were no significant differences in safety evaluation. These results suggest that these two preparations can replace each other during clinical use.

## Data Availability

The original contributions presented in the study are included in the article/Supplementary Material, further inquiries can be directed to the corresponding authors.
