# Supplementary material for: A single-dose, randomized, open-labeled, parallel-group study comparing the pharmacokinetics, pharmacodynamics and safety of leuprolide acetate microspheres 3.75 mg and Enantone® 3.75 mg in healthy male subjects
Source: Front Pharmacol. 2022 Aug 19;13:946505. doi: 10.3389/fphar.2022.946505 (PMC9437282; doi:10.3389/fphar.2022.946505)
Supplement: Supplementary file 1 [file Table1.DOCX]

Table S1. The quality control of quantitative analytical assays for leuprorelin in plasma, testosterone, LH, and FSH in serum.

| Analyte | Parameters | Normal | Precision (RSD, %) | Accuracy (%) |
| --- | --- | --- | --- | --- |
| Leuprorelin | LC-MS/MS method (Linear range: 0.0100-10.0 ng/mL) | | | |
|  | LLOQ, ng/mL | 0.0100 | 9.00 | 101.0 |
|  | LQC, ng/mL | 0.0300 | 7.99 | 99.33 |
|  | MQC1, ng/mL | 0.300 | 2.93 | 102.0 |
|  | MQC2, ng/mL | 5.00 | 2.29 | 102.2 |
|  | HQC, ng/mL | 7.50 | 2.76 | 102.3 |
| Testosterone | LC-MS/MS method (Linear range: 0.100-10.0 ng/mL) | | | |
|  | LLOQ, ng/mL | 0.100 | 6.43 | 99.40 |
|  | LQC, ng/mL | 0.300 | 3.99 | 102.0 |
|  | MQC, ng/mL | 4.00 | 1.80 | 102.2 |
|  | HQC, ng/mL | 7.50 | 2.31 | 101.2 |
|  | DQC (5x)△, ng/mL | 37.5 | 4.64 | 98.93 |
| FSH | Electrochemiluminescence immunoassay (Linear range: 0.111-162 mIU/mL) | | | |
|  | LLOQ, mIU/mL | 0.111 | 8.23 | 92.45 |
|  | LQC, mIU/mL | 0.288 | 5.36 | 91.32 |
|  | MQC, mIU/mL | 4.32 | 3.07 | 90.51 |
|  | HQC, mIU/mL | 130 | 1.91 | 102.7 |
| LH | Electrochemiluminescence immunoassay (Linear range: 0.0465-67.8 mIU/mL) | | | |
|  | LLOQ, mIU/mL | 0.0465 | 10.26 | 100.9 |
|  | LQC, mIU/mL | 0.120 | 4.05 | 95.00 |
|  | MQC, mIU/mL | 1.81 | 3.37 | 97.24 |
|  | HQC, mIU/mL | 54.2 | 3.14 | 97.05 |
|  | DQC (4x)△, mIU/mL | 90.3 | 3.15 | 96.90 |

Note: LLOQ, lower limits of quantification; LQC, low quality control; MQC, medium quality control; HQC, high quality control; DQC, dilution quality control. △DQC (5x or 4x), “5x” or “4x” means dilution factor.
